# Comparison of two methods in the treatment of congenital pseudarthrosis of clavicle: multicenter experience

**DOI:** 10.1186/s13018-021-02438-x

**Published:** 2021-05-08

**Authors:** Jin Li, Sheng Ping Tang, Hai Bo Mei, Jing Fan Shao, Bao Jie Shi, Hai Qiang Wang, Xin Tang, Chinese Multi-center Pediatric Orthopedic Study Group (CMPOS)

**Affiliations:** 1 Department of Orthopedics, Union Hospital, Tongji Medical College, Huazhong University of Science and Technology, Wuhan, 430022 China; 2 Department of Pediatric Orthopedics, Shenzhen Children’s Hospital, Shenzhen, 518046 China; 3 Department of Orthopedics, Hunan Children’s Hospital, Changsha, 410007 China; 4 Department of Pediatric Surgery, Tongji Hospital, Tongji Medical College, Huazhong University of Science and Technology, Wuhan, 430030 China; 5 Department of General Surgery, Xiang’an Hospital of Xiamen University, Xiamen, 361000 China; 6 Institute of Integrative Medicine, Shaanxi University of Chinese Medicine, Xixian Avenue, Xi’an, 712046 Xixian District China

**Keywords:** Congenital pseudarthrosis of clavicular, Surgical management, Iliac crest bone grafting, Elastic stable intramedullary nailing, K-wires, Plate

## Abstract

**Background:**

Congenital pseudoarthrosis of the clavicle (CPC) is an uncommon entity. Owing to its scarce presentation, treatment of this disorder has not been well established. This study aimed (1) to compare surgical treatment methods that included excision of pseudoarthrosis and iliac crest bone graft and fixate with either the elastic stable intramedullary nail (ESIN) or K-wires or plate and screws, and (2) to assess the clinical outcomes of two different surgical methods.

**Methods:**

A multi-central retrospective study was performed between 2013 and 2017 in four tertiary teaching hospitals. Fifteen clavicles of 11 children were identified as CPC. All patients underwent pseudarthrosis resection and iliac crest bone autograft. They were divided into two groups as per the surgical treatment they underwent—plate stabilization as group A and elastic stable intramedullary nailing (ESIN) or K-wires as group B. Nine clavicles in 6 patients in group A and 6 clavicles in 5 patients in group B, were included. The Quick Disabilities of the Arm and Shoulder (QuickDASH) score was used to assess patients’ satisfaction and function following treatment at each follow-up.

**Results:**

There were eight boys and three girls, with an average age of 4.7 years. All patients, except one with intellectual impairments, had radiological healing. Implant removal time was significantly shorter in group B compared to group A. No statistically significant differences existed in terms of age at surgery, time of radiological healing, complication, and clinical outcome between different groups.

**Conclusion:**

Surgical resection of pseudoarthrosis with an iliac crest bone graft was an effective means of surgical treatment in CPC. However, ESIN or K-wires can achieve shorter union time compared to the plate. Hence, surgical treatment is recommended for congenital pseudarthrosis of clavicular in pediatric patients.

**Level of evidence:**

Retrospective comparative study; Level III

## Introduction

Congenital pseudarthrosis of clavicular (CPC) is a rare disease, which was first reported in 1910 by Fitzwilliams. It has a predilection for the right clavicle in girls [1, 2], seldom occurred in the left side. Bilateral CPC occurs only in up to 10% of the cases according the literature [3, 4].

The etiology of CPC remains unclear; it is believed to be associated with the failure to fuse ossification centers of clavicle during the embryogenetic stage [5]. Another plausible hypothesis is related to the subclavian artery on the developing clavicle, which situates at a higher position than the normal during intrauterine life [3, 6]. Clinically, CPC usually presents a painless protuberance in the middle of the clavicle after birth or later in childhood. The deformity tends to be more evident with growth or some activities [4]. Surgical intervention is now widely accepted for the treatment of CPC. However, there is an obvious gap between basic and clinical research of CPC. This gap needs to be filled by emerging method and process such as translational orthopedics [7].

This multi-central retrospective study aimed to assess the clinical outcomes of different surgical methods in the treatment of CPC. This work may accelerate understanding more congenital pseudoarthrosis of the clavicle.

## Materials and methods

From 2013 January to 2017 December, all the cases of CPC who underwent surgical treatment were retrospectively reviewed at four high-volume, geographically separated, pediatric orthopedic centers. The inclusion criteria of the study were (1) pediatric patients aged < 14 years with a diagnosis of CPC, (2) undergoing surgical procedures and the iliac crest bone graft, (3) with a minimal follow-up of at least 24 months. Exclusion criteria were (1) follow-up less than 24 months, (2) suffer from cranial dysostosis, neurofibromatosis or other pathologic pseudarthroses of clavicular, (3) post-traumatic pseudarthrosis, and (4) incomplete medical records. The Ethics Committee of authors’ hospital approved the study. All guardians of these patients signed written informed consent.

All patients in the cohort underwent surgical treatment, including excision of the pseudarthrosis followed with implantation of iliac crest bone graft and stabilization with either ESIN or K-wires, or plates and screws. Details of surgical procedures were explained to the patient guardians and also explained the types of implants and let them choose. So, patients were divided into two groups as per the surgical treatment they underwent—plate stabilization as group A and elastic stable intramedullary nailing (ESIN) or K-wires as group B. Nine clavicles in 6 patients in group A and 6 clavicles in 5 patients in group B, were included. Postoperatively, all patients were advised to use a sling for 4-6 weeks. Implants were removed following radiological union or if unexpected complications occurred, such as deep infection or implants migration.

Hospital medical records and follow-up radiographs of each CPC patient were the main resources, including age at surgery, time to radiographic osseous consolidation, further surgery, implant removal time, and complications. Radiographic evaluation was taken at 4- or 6-week intervals before radiographic union, then change to 6- to 12-month intervals after implant removal. We used the Quick Disabilities of the Arm and Shoulder (QuickDASH) score to assess patients’ satisfaction and function after implant removal at final follow-up.

### Statistical analysis

Statistical analyses were performed using SPSS19.0 software (SPSS, IL, USA). Quantitative variables were expressed as mean ± standard deviation. Categorical variables were presented as values (percentages). A *p* value of < 0.05 is regarded as statistically significant at 95% confidence interval.

## Results

In total, 3 girls and 8 boys with 15 clavicles diagnosed as CPC were recruited. All patients had a painless lump without other subjective anomalies or disfunction, and all of them underwent the same surgical procedure using either ESIN or K-wires or plates and screws. The demographic and clinical data of the patients are illustrated in Tables 1 and 2.

**Table 1:** Demographic and data of patients in case series

	Sex	Side	Age at surgery (months/side)	Fixation (ESIN, K-wire, plate)	Radiological healing (months postoperative)	Removal of material (months postoperative)	Complication (yes=0, no=1)
1	M	B	141	ESIN		3 (L)	0
			141	ESIN		6 (R)	0
2	M	R	44	ESIN	3	5	1
3	M	R	23	ESIN	3	3	1
4	F	B	78 (L)	Plate	4	13	1
			84 (R)	Plate	4	7	1
5	M	R	66	K-wire	3	4	1
6	F	R	41	Plate	3	10	1
7	F	L	59	Plate	4	14	1
8	M	R	38	K-wire	6	6	1
9	M	B	45 (R)	Plate	4 (R)	12 (R)	1
			49 (L)	Plate	5 (L)	8 (L)	1
10	M	B	91 (R)	Plate	5	10	1
			91 (L)	Plate	5	10	1
11	M	R	36	Plate	4	9	1

*M* male, *F* female, *L* left, *R* right, *B* bilateral

**Table 2:** Summary of patients’ demographic and clinical characteristics

	Plate	ESIN or K-wires	*P* value
Age at surgery (months)	62.40± 46.58	58.33± 22.05	0.853
Radiological healing (months postoperative)	3.75± 1.50	4.00± 0.63	0.721
Removal of material (months postoperative)	4.60± 1.51	11.33± 1.97	**0.000**

**Fig. 1:**
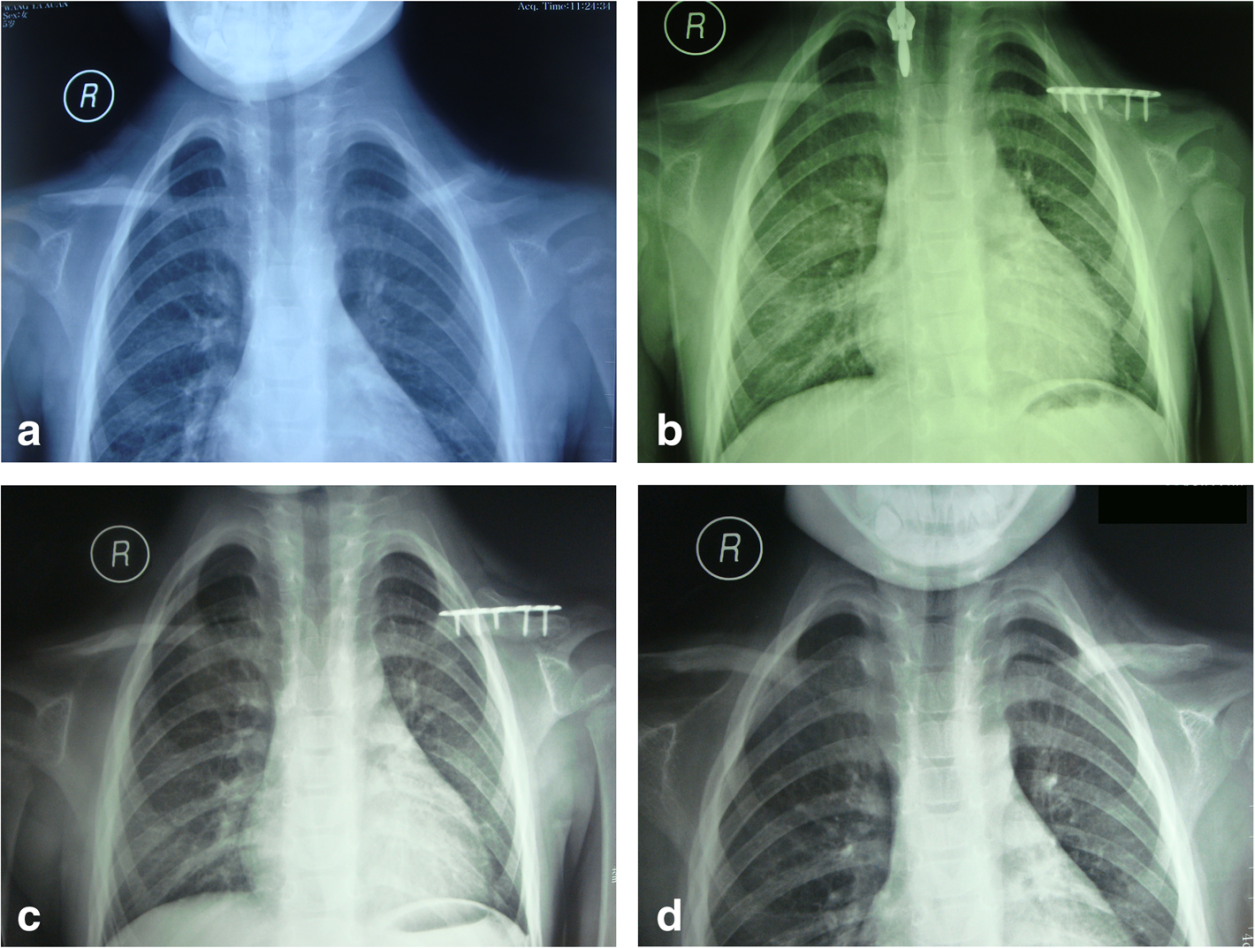
**a** Radiographic presentation of a 4.9-year-old girl with left clavicular pseudarthrosis preoperatively. **b** Postoperative radiograph with pseudarthrosis resection and iliac crest bone autograft stabilized with plate. **c** 11 months postoperative follow-up. **d** 8 months after removing the plate

In group A, 3 patients were males and 3 patients were females with a mean age at the time of surgery of 4.7 years. All the plates were locking plate. There were 3 patients with bilateral involvements, one was operated at the same time but the other two were 4- or 6-month intervals, whereas left side was in 1 and right side was in 2 patients. The mean follow-up period was 3 years. All patients achieved both clinical and radiological union at a median duration of 4.00± 0.63 months (range, 3-5 months). The average duration of implant removal was 11.33± 1.97 months postoperatively. The treatment and union process of a typical case shown in Fig. 1. All patients were satisfied with improved functional outcomes.

In group B, all 5 patients were males with a mean age at the time of surgery of 5.2 years. Only one patient was bilateral involvement, got operation at the same time, the other 4 patients were on the right side. The mean follow-up period was 3.5 years. Out of 5 patient, 4 patients with unilateral involvement achieved clinical and radiological union at a median duration of 3.75 ± 1.50 months (range, 3-6 months). The average duration of implant removal for these patients was 4.60 ± 1.51 months postoperatively.

**Fig. 2:**
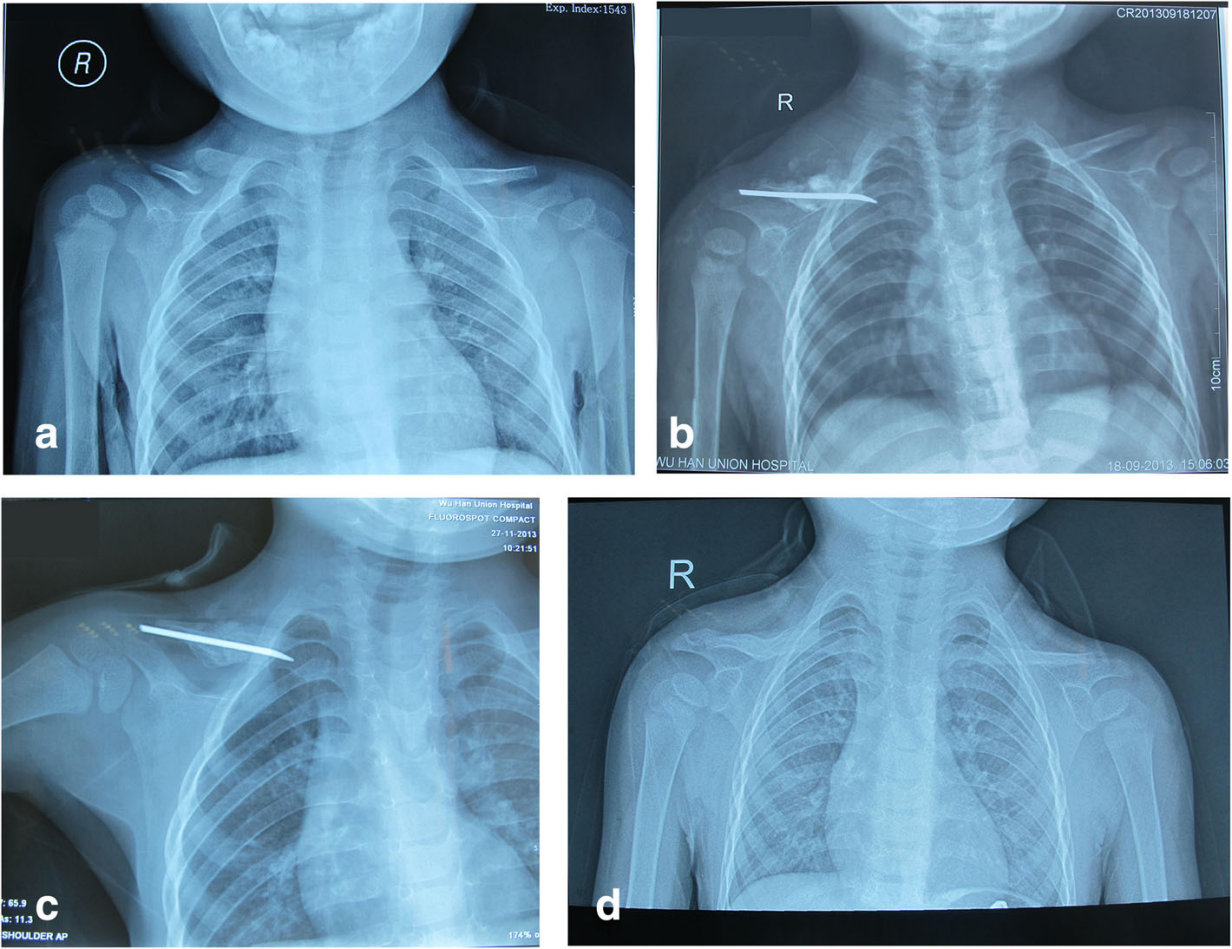
**a** Radiographic presentation of a 2-year-old boy with right-sided CPC preoperatively. **b** Postoperative radiograph with pseudarthrosis resection and iliac crest bone autograft stabilized with ESIN. **c** 2 months postoperative. **d** 6 months after removing the ESIN

However, the patient who had bilateral involvement failed to unite. The failure of union might attribute to postoperative complication, for the reason that he was lacking of treatment care for his intellectual impairments. He pulled out the left-sided pin at 3 months following surgery by himself and the right-sided pin was also migrated from the original site at 6 months follow-up. The pin was then removed, considering the risk of piercing the mediastinum, and the pseudarthrosis failed to heal. The treatment and union process of a typical case shown in Fig. 2. All patients, except 1 with bilateral involvement, were satisfied with improved functional outcomes.

Implant removal time was significantly shorter in group B compared to group A (*p*<0.001) (Table 2). No statistically significant differences existed in terms of age at surgery, time of radiological healing, complication, and clinical outcome. The QuickDASH score was 0 for all patients except the one whose pseudarthrosis failed to heal in group B.

## Discussion

Most important finding of the study is that both the surgical procedure provides satisfactory clinical and functional outcomes. However, patient selection is crucial as one of the patients with intellectual impairment in group B failed to unite postoperatively.

Congenital pseudarthrosis of the clavicle is an uncommon condition for which only over 200 cases have been reported [3, 8]. The malformation is more seen in females, mainly exists on the right side. Interestingly, both the genders were equally involved in this study, having more bilateral involvement cases (36%). The small sample size may have been responsible for this surprising result, and Persiani P. et al. also found no statistically significant difference between the girls and boys [9].

There were two opinions in the treatment of the CPC. The one is CPC should be treated conservatively. The other is surgical treatment was better for CPC. Considering the fact that the congenital pseudarthrosis of the clavicle causes only a few symptoms [10], a group of the authors preferred a conservative treatment [11, 12]. While surgical treatment is now progressively accepted due to increasingly obvious cosmetic deformity, pain, and functional disorders with growth [2, 3, 8]. Some authors considered the operative indication in a combination of cosmetic appearance and functional impairment. Pain and neurovascular symptoms such as thoracic outlet syndrome (TOS) also should be included [13]. Internal fixation was thought not necessary in children aged <3 years [14]. In this study, all the children and their parents asked for iliac crest bone graft and internal fixation in order to avoid increasingly obvious cosmetic deformity and unfavorable effect possibly.

All the cases in this study got autologous bone graft in operations. The use of bone graft has been strictly recommended in the literature, which leads to a higher percentage of success in union [15]. Meanwhile, using the autologous bone graft is preferable over allograft as of more beneficial biological characteristics. Iliac crest graft is the classic autologous bone graft. There were no successful reports of autologous bone graft substitution. Elliot et al. used a bovine cancellous xenograft to substitute iliac crest bone; however, it failed [8]. Another case of incorporating bone graft substitute leads to nonunion and a large defect [16]. According to the result of this study, autologous bone graft shows a higher percentage of the successful union, which was consistent with the literature.

The attitude toward surgical techniques is controversial. Di Gennaro GL et al. preferred the K-wire fixation when encountering septic nonunion using a plate fixation [17], but Toledo et al. reported an unstable smooth pin possibly result in a severe brachial plexus injury [11]. One study analyzes the treatment result of CPC by plate or K-wire and recommends internal fixation with Kirschner wires [9], whereas Chandran et al. reported that plate provides early union and with fewer complications compared to pin fixation (100% vs 60%) [18]. The stability may be one of the key points of bone union and avoiding complications. However, plate, ESIN, or K-wire did not show advantage of stability in this multi-central retrospective study. One patient in group B with bilateral involvement failed to unite. The reason for failure was because of wrong patient selection. As this male patient was suffering from intellectual impairment, and he pulled out the left pin at 3 months following surgery and the right pin was also migrated from the original site at 6 months follow-up. The pin was then removed, considering the risk of piercing the mediastinum, and the pseudarthrosis failed to heal. The failure could be prevented by the use of more rigid fixation like plate and screws in this patient. Another reason of failure was the age at operation. Some experts considered the best age was between 4 and 5 years [17–19]. If the age at operation was far beyond the best age, the difficulty of union will increase because the gap between bone graft and clavicle may need more time to heal. Indeed, the younger the child, the lesser symptoms may be present and more probability of splintering the bone. Surgeons should pay attention to the possibility of pin migration, which may be dangerous to the cardiovascular system. In addition to avoiding the ESIN or K-wires method, we also advise an early surgical age of CPC according with other authors.

Upper extremity function of most patients except one case stabilized with ESIN was normalized according to the QuickDASH score in this study. They all had a satisfactory prognosis accompanying with full range of pain-free movement at the shoulder and unrestricted activities and had no complications such as nonunion and infection. Implant removal for ESIN or K-wires group was significantly shorter than the plate group. This result was consistent with Lorente Molto and Currarino Guido’s study that it took much longer to remove plate (6 to 12 months) than pin (8 to 10 weeks) [4, 20].

Because of the rarity of the disease, this study was limited by small sample size. A large number of cases could have resulted in different results especially in patients’ demographics, surgical outcomes, and complications. Although all the patients achieved clinical and radiographic union, follow-up durations still varied. Lacking of randomization is another limitation of this study. As a retrospective multiple-center study with the patient guardians-chosen treatment, there were confounding factors even within same surgical treatments. Further studies with more patients and long-term follow-up are necessary.

## Conclusion

ESIN or K-wires seems to achieve quicker union compared to stabilization with plate in the treatment of CPC. K-wires should not be recommended considering the risk of migration into the medial structures, including the thoracic cavity and the great subclavian and other vessels. Surgical resection of pseudoarthrosis with an iliac crest bone graft and stabilization with plate was an effective means of surgical treatment in CPC, but need further research with more patients and long-term follow-up.

## Data Availability

The datasets used and/or analyzed during the current study are available from the corresponding author on reasonable request.

## Abbreviations

CPC: Congenital pseudarthrosis of the clavicle
ESIN: Elastic stable intramedullary nailing
K-wires: Kirschner wires
QuickDASH: Quick Disabilities of the Arm and Shoulder
TOS: Thoracic outlet syndrome
